# Are two plates better than one? A systematic review of dual plating for acute midshaft clavicle fractures

**DOI:** 10.1177/17585732211002495

**Published:** 2021-03-17

**Authors:** Ujash Sheth, Claire E Fernandez, Allison M Morgan, Patrick Henry, Diane Nam

**Affiliations:** 1 Sunnybrook Orthopaedic Upper Limb (SOUL), Division of Orthopaedic Surgery, Sunnybrook Health Sciences Centre, University of Toronto, Toronto, Canada; 2 Department of Orthopaedic Surgery, Northwestern University, Chicago, USA

**Keywords:** Clavicle fracture, precontoured plate, mini-fragment plate, hardware removal, reoperation

## Abstract

**Background:**

The rate of operative fixation of acute midshaft clavicle fractures has exponentially increased in recent years; however, the rate of reoperation for symptomatic hardware removal remains high and the optimal fixation strategy unknown. This systematic review aimed to summarize available evidence for dual plating of acute displaced midshaft clavicle fractures.

**Methods:**

EMBASE, MEDLINE, and PubMed searches identified clinical studies evaluating dual plate fixation of acute midshaft clavicle fractures. Pooled analysis was performed using a random-effects model in RevMan 5.3.

**Results:**

Eleven studies including 672 patients were included. Hardware removal occurred in 4.4% and 12.3% of patients undergoing dual and single plate fixation, respectively. Compared to single plating, dual plating had significantly lower odds of hardware removal (*P* = 0.001) with no difference in union rates. There were no significant differences in reoperation (excluding hardware removal), complications, and patient-reported outcomes between the two groups (*P* > 0.05).

**Conclusions:**

This study suggests that dual plating of acute displaced midshaft clavicle fractures may lead to lower rates of reoperation for symptomatic hardware removal without compromising fracture healing. Ultimately, well-designed randomized trials are needed to further investigate the findings from this systematic review.

## Introduction

Midshaft clavicle fractures have traditionally been treated non-operatively with immobilization in a sling or a figure-of-eight bandage.^1,2^ Early studies reported a non-union rate of 1% and negligible functional consequence with conservative management. However, the inclusion of pediatric fractures and an absence of modern functional assessments in these studies resulted in an underestimation of non-union rates and overly optimistic clinical outcomes.^1–3^ More recent data have demonstrated a non-union rate of 15% with approximately 30% of patients dissatisfied with their outcome following non-operative treatment.^4–8^ As a result, there has been renewed interest in surgical fixation of displaced midshaft clavicle fractures. In fact, the rate of operative fixation has exponentially increased following the publication of a landmark randomized controlled trial by the Canadian Orthopaedic Trauma Society which was the first of many level I studies to demonstrate higher union rates, decreased rates of symptomatic malunion, earlier return to function, and improved patient-reported outcomes with plate fixation of displaced midshaft clavicle fractures.^6,9–11^

Traditional plating techniques for open reduction and internal fixation of clavicle fractures involve the use of a single 3.5-mm plate placed superiorly or anteriorly.^12^ However, these plates are often very prominent under the skin causing irritation, and in many cases result in reoperation for hardware removal. The rate of reoperation for removal of symptomatic hardware has been reported to range from 8% to 66%.^10,13–20^ For this reason, there have been a number of different fixation strategies described to minimize the need to return to the operating room for implant-related symptoms.^21^ These include intramedullary nailing^22,23^ and the use of anatomic precontoured clavicle plates positioned superiorly^24,25^ or anteroinferiorly.^26–28^

More recently, a dual plate construct using two mini-fragment plates placed orthogonally has been advocated as a means of decreasing the rate of reoperation for symptomatic hardware removal. In 2015, Prasarn et al.^29^ reported on a series of 17 patients undergoing clavicle fixation using a 2.7-mm plate positioned superiorly and a 2.4-mm plate positioned anteriorly and had no reoperations while noting a 100% union rate. The idea of dual plating is not new^30–31^ and is commonly used in the setting of clavicle fracture non-union fixation.^32,33^ Its efficacy has also been described in distal clavicle fractures.^34–38^ Biomechanical studies investigating dual plating versus single plating specifically for midshaft clavicle fracture have mostly found dual plating to be equivalent to single plating, though this finding is not unanimous. In their cadaveric study, Ziegler et al. found no significant differences between a single 3.5-mm anteroinferior plate, a single 3.5-mm superior plate, and dual 2.7-mm plates in axial stiffness, bending stiffness, torsional stiffness, or bending load to failure.^39^ Prasarn et al.^29^ utilized a sawbones model to similarly conclude that dual plating was biomechanically equivalent to single plating. Furthermore, a recent finite element analysis by Zhang et al.^40^ found no significant difference in cantilever bending, axial compression, and axial torsion between single and dual plate constructs, while also noting the highest stiffness and least micromotion with dual plate fixation. However, a recent study by Boyce et al. cautioned against conclusions of equivalence between dual and single plate constructs, finding that dual fixation demonstrated lower stiffness and strength than single fixation in their sawbones model.^41^ Of note, all such biomechanical studies are limited by a lack of knowledge on the minimum strength truly required for clavicle fixation in vivo. Dual plating may serve as an alternative to a single precontoured plate with a biomechanically similar or equivalent profile and the additional benefit of being a lower profile implant to which may help diminish the high rate of symptomatic implant removal observed with single plating.

The objective of this systematic review is to summarize the available clinical evidence for dual plating of acute displaced midshaft clavicle fractures. An evaluation of the available clinical outcomes including comparisons to conventional plating techniques (i.e. single precontoured plate) will allow for the assessment of the safety and efficacy of dual plating.

## Materials and methods

This systematic review was conducted following the Preferred Reporting Items for Systematic Reviews (PRISMA) guidelines.^42^

### Literature search

The electronic databases EMBASE, MEDLINE, PubMed, and Google Scholar were searched for articles available as of 6 June 2020. A title, abstract, and full-text screen were performed to identify relevant articles. The following search terms were used: “dual” or “double” or “mini-frag*” or “mini plates” or “semi-tubular plates” or “tubular plates” and “clavic*” or “fracture” and “clavic*”. This search was limited to the English language. References of included studies were reviewed for additional relevant articles that met the inclusion criteria.

### Eligibility criteria

Studies were included if they addressed acute, midshaft clavicle fractures managed with dual plate fixation and were published in English. Technical and clinical studies were included. Restrictions for years of publication were not deemed necessary. Studies were excluded if they discussed dual plate fixation in the setting of distal clavicle fracture or non-union. Review articles and expert opinions were also excluded. Studies with small sample size (i.e. less than 10) or case reports were included to increase the pool of data. 

### Study selection

Two independent reviewers (CEF and AMM) screened the titles and abstracts of all identified articles for eligibility. Duplicate articles were manually excluded. Both reviewers evaluated the full text of all potentially eligible studies identified by title and abstract screening to determine final eligibility. Disagreements were resolved by a consensus decision in conjunction with the senior author.

### Data extraction

Data were extracted independently and in duplicate by both reviewers. Data collected from all relevant studies included: author, year of publication, title, journal, level of evidence, sample size, sex and age of participants, surgical technique, length of follow-up, union rate, complications, reoperations, patient-reported outcomes, and other notable results.

### Assessment of performed risk of bias in eligible studies

Two reviewers (CEF and AMM) performed an independent assessment of the methodological quality of each eligible study using the Methodological Index for Non-Randomized Studies (MINORS). The MINORS criteria is a validated 12-item instrument used to evaluate the methodological quality of non-randomized (*non-comparative* and *comparative*) surgical studies. Items are scored as 0 (not reported), 1 (reported but inadequate), or 2 (reported and adequate). The maximum global score for non-comparative and comparative studies was 16 and 24, respectively.^43^

### Statistical analysis

Descriptive statistics were calculated with categorical data presented as frequency with percentages and continuous data as a mean ± standard deviation (SD). Weighted means were calculated for all parameters. Mean differences were calculated for continuous outcomes and odds ratios (ORs) for dichotomous outcomes. Ninety-five percent confidence intervals (CIs) were reported for all point estimates. A random-effects model was used for pooled comparisons. Pooled estimates were calculated using Review Manager 5.3.^44^

## Results

### Study characteristics

**Figure 1.**
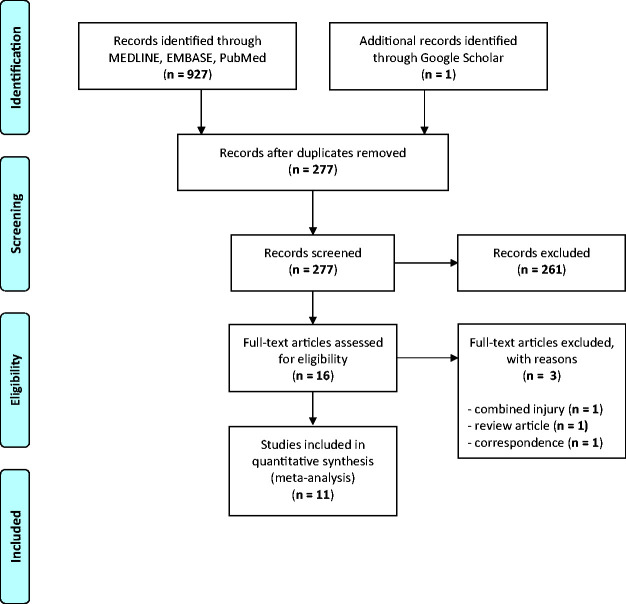
PRISMA flow diagram.

The initial search yielded 928 articles, of which 650 were duplicates. Following application of inclusion and exclusion criteria, 11 articles were included in this systematic review^29–31,39,40,45–52^ (Figure 1). A summary of included studies is demonstrated in Table 1. Among the 11 clinical studies, there were a total of 672 patients, including 389 who underwent dual plating and 283 who had single plate fixation of their acute midshaft clavicle fracture. The majority of patients were male (78.2%) with a mean age of 37.2 years. The mean follow-up across all clinical studies was 23.2 months.

**Table 1.** Summary of clinical studies.

Study	Sample Size, *N*		Mean Age, *years* (SD)		Male Sex, *N* (%)		Mean follow-up, *months* (SD)	Outcome measures
	Single	Dual	Single	Dual	Single	Dual		
Allis et al.^45^	21	23	36 (14.0)	36 (12.0)	NR	NR	39.2 (16.1)	Union rate, reoperation rate, ASES score
Chen et al.^46^	125	34	39.7 (15.2)	39.1 (18.1)	96 (76.8)	28 (82.0)	9.6 (4.8)	Union rate, complications, reoperation rate
Chen et al.^47^	–	36	–	40.6 (15.3)	–	24 (66.7)	23.5 (41)	Union rate, complications, reoperation rate, QuickDASH score
Czajka et al.^48^	–	81	–	31.3 (8.3)	–	63 (77.8)	15.7 (8.5)	Reoperation rate, QuickDASH score
DeBaun et al.^49^	74	60	44 (14)	44 (16)	60 (81.1)	43 (71.7)	9.0 (7.5)	Union rate, complications, reoperation rate
Giordano et al.^50^	–	1	–	23 (N/A)	–	1 (100)	12 (N/A)	Union rate
Lee et al.^51^	33	89	30.6 (10.3)	28.9 (10.4)	77 (86.5)	28 (85)	24 (NR)	Union rate, complications, reoperation rate, operating time
Prasarn et al.^29^	–	17	–	31.3 (12.8)	–	15 (88.2)	16.1 (6.5)	Union rate, reoperation rate, DASH score
Qamar et al.^30^	–	20	–	39.2 (11.0)	–	15 (75)	36 (10.5)	Union rate, complications, reoperation rate, DASH score
Shannon et al.^31^	–	13	–	41.2 (18.1)	–	12 (92.3)	22.3 (11.8)	Union rate
Zhuang et al.^52^	30	17	37.0 (12.2)	39.3 (13.6)	17 (56.7)	12 (70.6)	10.3 (3.5)	Union rate, Constant-Murley score

N: count; SD: standard deviation; NR: not reported; ASES: American Shoulder and Elbow Score; DASH: Disabilities of the Arm Shoulder and Hand; N/A: not applicable.

### Quality assessment

An assessment of the methodological quality of eligible studies was performed using the MINORS criteria (Supplemental File 1). The mean scores for non-comparative and comparative studies were 9.7 (out of 16) and 19 (out of 24), respectively.

### Surgical technique

#### Plating construct

Various implants were used for dual and single plate fixation across studies (Table 2). Implant choice was based on surgeon preference and construct availability. In most cases, dual plating consisted of a 2.0/2.4/2.7-mm plate superiorly and a 2.0/2.4/2.7-mm plate anteriorly or anteroinferiorly.^29,39,45–51^ Zhuang et al. used a 3.5-mm locking compression plate or reconstruction plate superiorly and a mini-fragment placed anteriorly.^52^ Two studies used one-third tubular plates,^30,31^ while Chen et al. used a combination of one-third tubular, locking, and reconstruction plates.^46^

**Table 2.** Summary of plate constructs used.

Study	Dual plate construct	Single plate construct
Allis et al.^45^	2.7-mm superior and 2.4-mm anterior mini-fragment plates	3.5-mm precontoured clavicle plate (superior or anterior)
Chen et al.^46^	Tubular and locking compression plate OR tubular and reconstruction plate OR locking compression and reconstruction plate	Superior/anteroinferior locking compression or reconstruction plate
Chen et al.^47^	2.4/2.4-mm or 2.0/2.4-mm superior and anterior mini-fragment plates	–
Czajka et al.^48^	2.7-mm superior and 2.4-mm anterior mini-fragment plates	–
DeBaun et al.^49^	2.7 -, 2.4 -, or 2.0-mm superior and anterior mini-fragment plates	3.5-mm precontoured clavicle plate (superior or anterior)
Giordano et al.^50^	2.3/2.3-mm superior and anterior mini-fragment plates	–
Lee et al.^51^	2.7/2.7-mm superior and anterior reconstruction plates	3.5-mm locking compression plate
Prasarn et al.^29^	2.7-mm superior and 2.4-mm antero-inferior mini-fragment plates	3.5-mm superior or antero-inferior locking reconstruction plate
Qamar et al.^30^	Two 3.5-mm one-third semi-tubular plates	–
Shannon et al.^31^	One-third tubular, one-quarter tubular or 2.0-mm superior or anterior mini-fragment plates with 2.7-mm or 3.5-mm pelvic reconstruction plates or 3.5-mm superior locking compression plate	–
Zhuang et al.^52^	3.5-mm superior locking compression plate or reconstruction plate and anteroinferior mini-fragment “aid plate”	3.5-mm antero-inferior locking compression plate

### Clinical outcomes

#### Non-union

**Figure 2.**
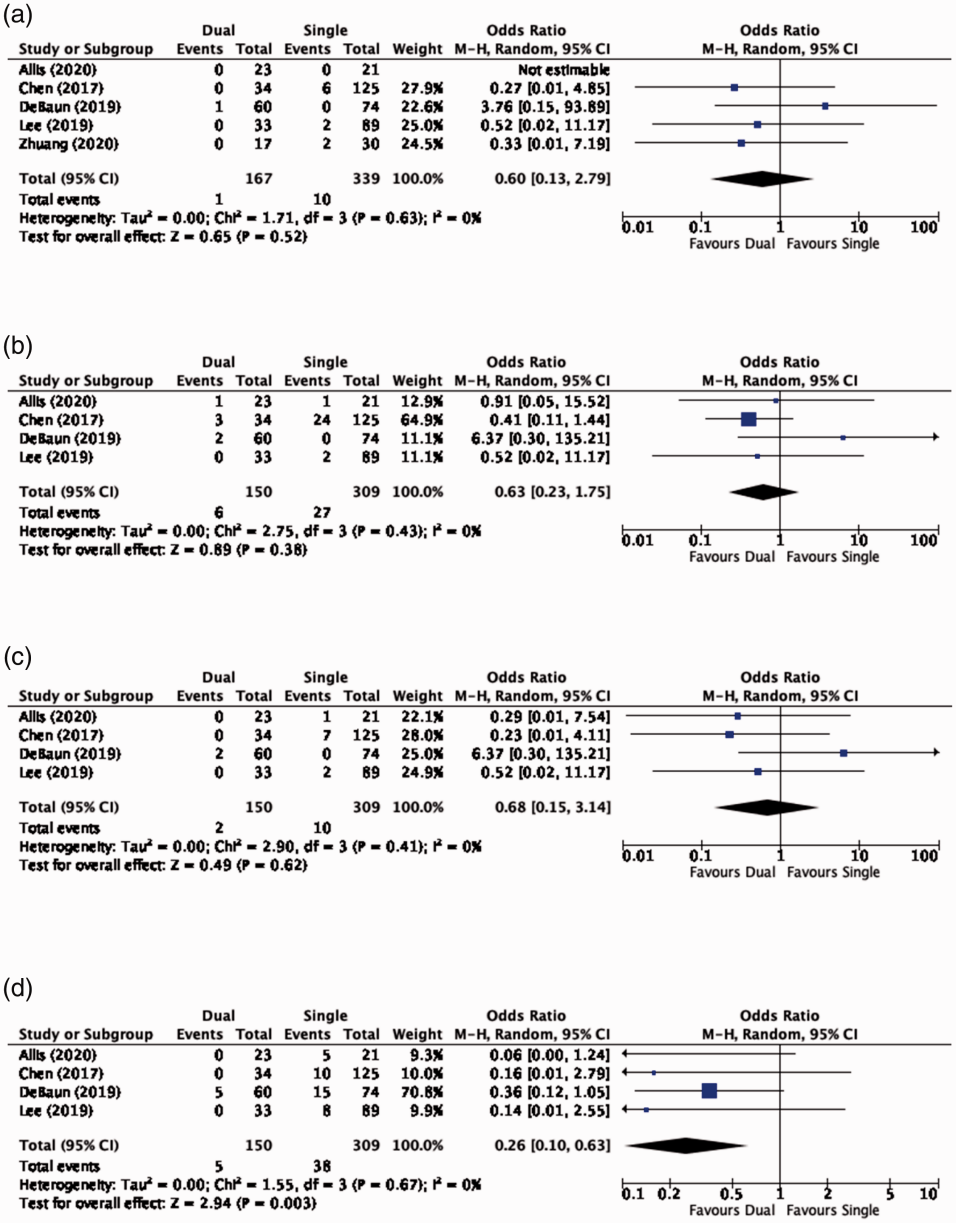
Clinical outcomes. (a) Non-unions. (b) Complications. (c) Reoperations excluding hardware removal. (d) Hardware removal.

Non-union was reported in 10 studies.^29–31,45–47,49–52^ The overall non-union rate was 3.4%, with 0.8% (*N* = 254) of dual plated and 2.9% (*N* = 339) of single plated clavicles going on to non-union. Pooled comparison across the five comparative studies^45,46,49,51,52^ demonstrated no significant difference in the odds of fracture non-union with dual and single plate fixation methods (OR, 0.60; 95% CI, 0.13 to 2.79; *P* = 0.52) (Figure 2(a)).

#### Delayed union

The incidence of delayed union was noted in two comparative studies.^46,51^ The rate of delayed union among patients with a single plate construct was 3.7% (*N* = 214), while no case of delayed union was noted among the dual plated group.

#### Complications

Complications were described in 10 studies^29–31,45–51^ and included intraoperative and postoperative complications such as neurovascular injury and infection. Complications were defined in a heterogenous manner across studies, as such, we defined a complication as an unintended negative outcome other than non-union, delayed union, and reoperation, which were reported separately in this review.

The overall complication rate was 5.9%. The complication rate in the dual and single plate groups were 3.1% (*N* = 318) and 8.7% (*N* = 309), respectively. Pooled analyses of the four comparative studies^45,46,49,51^ found no significant difference in the odds of a complication occurring with dual and single plate constructs (OR, 0.63; 95% CI, 0.23 to 1.75; *P* = 0.38) (Figure 2(b)).

#### Reoperation

Reoperation excluding hardware removal was reported in nine clinical studies.^29–31,45,46,48,49,51,53,54^ Indications for reoperation included non-union, mal-union, and deep infections. The overall reoperation rate across all studies was 2.4%. The reoperation rate for dual and single plate fixation was 1.3% (*N* = 237) and 3.2% (*N* = 309), respectively. Pooling data across the four comparative studies^45,46,49,51^ revealed no significant difference in the odds of reoperation for causes other than symptomatic hardware removal when comparing dual and single plate constructs (OR, 0.68; 95% CI, 0.15 to 3.14; *P* = 0.62) (Figure 2(c)).

#### Hardware removal

A total of 10 studies provided data on hardware removal.^29–31,45–51^ The overall rate of hardware removal was 8.3%. Hardware removal occurred in 4.4% (*N* = 318) and 12.3% (*N* = 309) of patients undergoing dual and single plate fixation, respectively. Among the four comparative studies,^45,46,49,51^ dual plating resulted in a 77% lower odds of hardware removal compared to single plate fixation (OR, 0.23; 95% CI, 0.07 to 0.78; *P* = 0.02) (Figure 2(d)).

#### Patient-reported outcomes

Patient-reported outcome measures included Disabilities of the Arm, Shoulder, and Hand (DASH), a shortened version of this score known as the QuickDASH, the American Shoulder and Elbow Surgeon Score (ASES), and the Constant-Murley Score. The DASH score was reported in four studies^29,30,47,48^ (*N* = 35) with an overall weighted mean score of 6.5 ± 6.6 at final follow-up. The QuickDASH score was collected in two case series^48,53^ (*N* = 101) and found to have an overall weighted mean score of 7.8 ± 11.8. Allis et al.^45^ reported no difference in ASES scores between patients undergoing clavicle fracture fixation using a single 3.5-mm precontoured clavicle plate versus dual mini-fragment plates (*P* = 0.14). When comparing the Constant-Murley score between single and dual plate groups at three and six months, Zhuang et al.^52^ noted a significant difference favoring dual plating at three months postoperatively (*P* = 0.002); however, the difference did not persist at six months (*P* = 0.054).

### Implant cost

Czajka et al.^48^ found the mean implant cost in 2016 of a dual mini-fragment plate construct for midshaft clavicle fracture fixation to be $1511.38 USD compared to $1253.08 USD for a single, 3.5-mm precontoured plate from the same manufacturer (Synthes, Paoli, PA). However, Qamar et al. in their 2011 publication^30^ concluded that their dual plate construct (using two one-third tubular plates) would be less expensive than a similar construct using a locking compression plate with locking screws or a precontoured clavicle plate. The authors note that although the cost of a locking compression plate is similar to two one-third semi-tubular plates, the locking screws are eight times more expensive than conventional cortical screws, while the precontoured clavicle plate itself is 10 times more expensive than two one-third tubular plates. Of note, this study came out of the United Kingdom and further information beyond relative costs, i.e. exact amounts and currency referenced, was not discussed.^30^

### Operative time

The mean operative time for dual plating was reported in two studies^48,51^ and varied from 97 min (SD 12) to 174 min (SD 45). Lee et al.^51^ was the only study to directly compare operative times between dual and single plate constructs and found a significant difference in mean operative time favoring single plating by nearly 1 h (mean difference 55 min; 95% CI, 39 to 71; *P < *0.001).

## Discussion

Despite variations in implant choice and plating construct, the current systematic review demonstrates that the use of dual orthogonal plating of acute displaced midshaft clavicle fractures may result in lower rates of reoperation for hardware removal compared to single plate fixation without compromising union rates. Moreover, the rate of non-union and complication was two to three times higher among single plate constructs; however, there was no statistically significant difference between single and dual plating.

Although plate fixation of displaced midshaft clavicle fractures has led to lower non-union rates, earlier return to work, and better function than non-operative treatment, the high rates of reoperation due to plate prominence and hardware irritation remain an ongoing concern. The decision to undergo hardware removal is a subjective choice made between patient and surgeon. Although no standardized indications or objective measurements to guide this decision are currently described in the literature, symptomatic hardware removal is frequently reported. The introduction of precontoured clavicle plates has reduced the rate of symptomatic hardware removal, especially when plating anteroinferiorly.^15,20,55^ However, even with the use of precontoured plates, the reported hardware removal rate varies from 5% to 47%.^55,56^ This is likely due to the significant variation in clavicle anatomy (i.e. sigmoid curve, coronal bow, and length) observed between individuals which precludes anatomic fitting of precontoured plates in all patients. In fact, Malhas et al.^57^ published a cadaveric study that found further contouring of precontoured plates was necessary in 73% of cases to optimize plate–bone fit. In contrast, dual mini-fragment plates are lower profile and offer the advantage of precisely contouring the plates to fit individual patient anatomy.^47^

The benefits associated with a dual plate construct extend beyond a reduction in implant-related soft-tissue irritation. Intraoperatively, dual plating allows for (1) more points of fixation, (2) buttressing of anterior butterfly fragments, (3) mini-fragment plates to be used as washers for multiple lag screws, and (4) the use of either the superior or anterior plate as a reduction aid or clamp while the second plate is applied.^29^ Based on prior biomechanical data, the ability of a dual plate construct to withstand multiplanar bending forces better than a single plate construct may allow for the theoretical advantage of early weight-bearing through the affected extremity.^29,40^ In fact, Czajka et al.^48^ allowed immediate unrestricted range of motion post-operatively and unrestricted weight-bearing at six weeks in a cohort of patients who underwent dual plate fixation of displaced midshaft clavicle fractures.

One of the most significant concerns regarding dual plating techniques has been the theoretical risk of non-union due to compromised vascularity from the soft tissue stripping required to place two orthogonal plates on the clavicle. However, the use of an extraperiosteal exposure results in a minimal increase in soft tissue stripping for application of a second mini-fragment plate.^31,46^ Moreover, the non-union rate following dual plate fixation in the current systematic review was less than 1%, which suggests that fracture healing is not compromised with application of dual orthogonal plates.

A potential barrier to the routine use of a dual plate construct for fixation of displaced midshaft clavicle fractures may be the additional operative time required. Czajka et al.^48^ hypothesized that dual plating would require more surgical time due to the increased soft tissue dissection required for exposure and the time required for contouring and application of two plates. However, operative times varied significantly between and within the two studies that reported these data in our review.^48,51^ For instance, four different surgeons performed dual plating in the study by Lee et al.^51^ with one surgeon’s operative time averaging 55 min longer than the others. There has also been a wide range of reported operative times for single plate fixation, with a mean operative time of 65–80 min (range 35–179 min) noted in the literature.^58–60^ These differences make it difficult to discern if operative time is truly dependent on the plating construct or the individual surgeon. Moreover, dual plating may be more technically challenging due to the need to contour plates to bone with a sinusoidal shape, which has its own learning curve.

The costs associated with a dual plate construct varied greatly between studies secondary to the implant used. Among the two studies that examined costs, Qamar et al.^30^ used two 3.5-mm one-third tubular plates, which was a significantly cheaper construct than a precontoured clavicle plate. Meanwhile, Czajka et al.^48^ used two mini-fragment plates, which was approximately $300 more expensive than the precontoured plate. However, the cost of mini-fragment and precontoured plates has recently been shown to vary by as much as $1900 based on vendor.^61^ Despite this variation in implant cost, it still remains to be seen whether dual plating is a cost-effective long-term strategy. However, it stands to reason that the potential cost-savings from decreased reoperation rates of symptomatic hardware removal would also result in greater benefit to the patient with earlier and uninterrupted return to baseline activities.

The current review has a number of limitations. Firstly, there were very few studies directly comparing single and dual plate constructs, as such, this review was limited to level III and IV studies. Secondly, follow-up intervals varied considerably across studies ranging from 6 to 39 months, which may have an effect on the development of symptomatic hardware. However, the majority of studies did have greater than one year of follow-up. Thirdly, multiple surgeons often performed dual plate fixation in each study, which contributed to the wide range of operative times reported. Despite the use of various dual plating constructs, all included studies were published after 2011, which indicates that modern plating techniques were used. Finally, implant removal is typically a subjective decision between the patient and surgeon, as such, it is subject to multiple biases. There are no standardized indications for hardware removal nor objective measures reported to elucidate how these decisions are made, which remains an important limitation of the existing literature. Despite these limitations, the outcome measures used were homogenous across the 11 included studies which allowed for pooling of common outcome measures across comparative studies.

## Conclusion

Based on the current literature, dual plating of acute displaced midshaft clavicle fractures may lead to lower rates of reoperation for symptomatic hardware removal without compromising healing. However, further biomechanical and clinical studies are warranted to determine the optimal implant choice (e.g. 2.0 mm versus 2.7 mm), configuration (i.e. plate placement superior/anterior), and cost-effectiveness of dual plating constructs. Ultimately, large, well-designed, randomized trials are needed to further investigate the findings from this review.

## Supplemental Material

sj-pdf-1-sel-10.1177_17585732211002495 - Supplemental material for Are two plates better than one? A systematic review of dual plating for acute midshaft clavicle fractures Click here for additional data file. Supplemental material, sj-pdf-1-sel-10.1177_17585732211002495 for Are two plates better than one? A systematic review of dual plating for acute midshaft clavicle fractures by Ujash Sheth, Claire E Fernandez, Allison M Morgan, Patrick Henry and Diane Nam in Shoulder & Elbow
