# Supplementary material for: Are two plates better than one? A systematic review of dual plating for acute midshaft clavicle fractures
Source: Shoulder Elbow. 2021 Mar 17;14(5):500–9. doi: 10.1177/17585732211002495 (PMC9527487; doi:10.1177/17585732211002495)
Supplement: sj-pdf-1-sel-10.1177_17585732211002495 - Supplemental material for Are two plates better than one? A systematic review of dual plating for acute midshaft clavicle fractures [file sj-pdf-1-sel-10.1177_17585732211002495.pdf]

Supplemental File 1. Methodological Quality of Eligible Studies Based on MINORS Criteria

Study (Year)	Clearly stated aim	Inclusion of consecutive patients	Prospective collection of data	Endpoint appropriate to the aim of the study	Unbiased assessment of the study endpoint	Follow-up period appropriate to the aim of the study	Loss of follow-up less than 5%	Prospective calculation of the study size	Additional Criteria for Comparative Studies				
									Adequate control group	Contemporary group	Baseline equivalence of groups	Adequate statistical analysis	Total (/24)* (/16)^
Allis (2020)	2	2	1	2	0	2	1	0	2	2	2	2	**18**
Chen (2017)	2	2	1	2	0	2	2	0	2	2	2	2	**19**
Chen (2020)	2	2	1	2	0	1	0	0					**8**
Czajka (2017)	2	2	1	2	0	2	1	0					**10**
DeBaun (2019)	2	2	1	2	0	1	2	2	2	0	1	2	**20**
Giordano (2018)	2	1	0	2	0	2	2	0					**9**
Lee (2019)	2	2	1	2	0	2	1	2	2	2	2	2	**20**
Prasarn (2015)	2	2	1	2	0	2	1	0					**10**
Qamar (2011)	2	2	1	2	0	2	1	0					**10**
Shannon (2016)	2	2	1	2	0	2	2	0					**11**
Zhuang (2020)	2	2	1	2	0	1	2	0	2	2	2	2	**18**

*Maximum possible score for *comparative* studies

^Maximum possible score for *noncomparative* studies
